# Plant Extracts Containing Saponins Affects the Stability and Biological Activity of Hempseed Oil Emulsion System

**DOI:** 10.3390/molecules25112696

**Published:** 2020-06-10

**Authors:** Maciej Jarzębski, Przemysław Siejak, Wojciech Smułek, Farahnaz Fathordoobady, Yigong Guo, Jarosław Pawlicz, Tomasz Trzeciak, Przemysław Łukasz Kowalczewski, David D. Kitts, Anika Singh, Anubhav Pratap Singh

**Affiliations:** 1 Department of Physics and Biophysics, Faculty of Food Science and Nutrition, Poznań University of Life Sciences, Wojska Polskiego 38/42, 60-637 Poznań, Poland; maciej.jarzebski@up.poznan.pl (M.J.); przemyslaw.siejak@up.poznan.pl (P.S.); 2 Institute of Chemical Technology and Engineering, Poznan University of Technology, Berdychowo 4, 60-695 Poznan, Poland; 3 Food Nutrition and Health Program, Faculty of Land and Food Systems, The University of British Columbia, 2205 East Mall, Vancouver, BC V6T 1Z4, Canada; farah.fathordoobady@ubc.ca (F.F.); yigong.guo@ubc.ca (Y.G.); david.kitts@ubc.ca (D.D.K.); anika.singh@ubc.ca (A.S.); 4 Department of Orthopedics and Traumatology, Poznan University of Medical Sciences, 28 Czerwca 1956 135/147, 61-545 Poznań, Poland; jarekpawlicz@gmail.com (J.P.); trzeciak@orsk.ump.edu.pl (T.T.); 5 Institute of Food Technology of Plant Origin, Poznań University of Life Sciences, Wojska Polskiego 31, 60-624 Poznań, Poland; przemyslaw.kowalczewski@up.poznan.pl

**Keywords:** hempseed oil, *Quillaja saponaria*, *Saponaria officinalis*, nanoemulsion, particle size, dynamic light scattering

## Abstract

In this study, two saponins-rich plant extracts, viz. *Saponaria officinalis* and *Quillaja saponaria*, were used as surfactants in an oil-in-water (O/W) emulsion based on hempseed oil (HSO). This study focused on a low oil phase content of 2% *v*/*v* HSO to investigate stable emulsion systems under minimum oil phase conditions. Emulsion stability was characterized by the emulsification index (EI), centrifugation tests, droplet size distribution as well as microscopic imaging. The smallest droplets recorded by dynamic light scattering (droplets size v. number), one day after the preparation of the emulsion, were around 50–120 nm depending the on use of *Saponaria* and *Quillaja* as a surfactant and corresponding to critical micelle concentration (CMC) in the range 0–2 g/L. The surface and interfacial tension of the emulsion components were studied as well. The effect of emulsions on environmental bacteria strains was also investigated. It was observed that emulsions with *Saponaria officinalis* extract exhibited slight toxic activity (the cell metabolic activity reduced to 80%), in contrast to *Quillaja* emulsion, which induced *Pseudomonas fluorescens ATCC 17400* growth. The highest-stability samples were those with doubled CMC concentration. The presented results demonstrate a possible use of oil emulsions based on plant extract rich in saponins for the food industry, biomedical and cosmetics applications, and nanoemulsion preparations.

## 1. Introduction

Fats and oils are key elements of the human diet, providing essential fatty acids [1,2] which are often employed as raw materials for food production. The growing awareness about the health benefits and nutritional properties of hempseed oil (HSO) from industrial hemp (*Cannabis sativa* L.) has encouraged recent researchers to focus on the innovative application of this oil. The balanced ratio of omega-6 to omega-3 fatty acids (from 2.1 to 3.1) in HSO is considered optimal for human health and triggers several physiological reactions in the body, including vasodilation, pro-aggregator, and inflammatory responses [3,4]. The presence of γ-linolenic acid, stearidonic acid (18:4, *n*-3), terpenoids, β-sitosterol, as well as antioxidants, including phenolics and flavonoids, depicts the potential of this oil for application in oil-based food and pharmaceutical formulations [4,5]. Small quantities of non-psychoactive cannabinoids such as cannabidiol and cannabigerol in hempseed oil have been documented to exhibit anti-inflammatory, anti-nausea, anti-convulsive, and anti-epileptic effects [6]. HSO has also shown antimicrobial activity against some bacteria including *Bacillus subtilis*, *Staphylococcus aureus*, *Micrococcus luteus* as well as *Escherichia coli* [7,8]. Moreover, showing both hydrophilic and hydrophobic properties make HSO suitable as the oil phase for various emulsion systems.

McClements defined an oil-in-water (O/W) nanoemulsion as a thermodynamically unstable colloidal dispersion, consisting of two immiscible liquids where the dispersed droplets have spherical shape [9]. Droplet size has been proposed as one way of differentiating between microemulsions and nanoemulsions, wherein emulsions with a droplet radius (R) smaller than 100 nm are often classified as nanoemulsions [10]. Nanoemulsions are commonly prepared by dispersing the two immiscible liquids using high-energy mixing equipment, such as high-speed mixers and homogenizers [11]. Surfactants or emulsifiers are often used to increase emulsion stability. The type of surfactant and its physicochemical properties, including critical micelle concentration (CMC) and hydrophilic–lipophilic balance (HLB), play an important role in emulsion stabilization [12]. Surface-active compounds are often supported by co-surfactants, which increase nanoemulsion stability [13]. Various nanoemulsion systems have been investigated due to their long-term stability and cellular uptake efficiency [14] for possible use in the food industry, medicine, pharmaceutics and cosmetics [15]. Specifically, for food and biomedical applications [16], nanoemulsion systems were considered as carriers [17], which allows for incorporating various compounds, such as active or bioactive agents [18], food additives [19], dyes, etc. Oil-based nanodroplets can be considered as a new delivery system for bioactive food compounds such as vitamins [20]. Using chemical-based surfactants has raised lots of health concern [21]. For that reason, developing natural surfactancs, including plant surface-active compounds such as saponins, can be considered as an alternative for food and biomedical nanoemulsion applications [22].

Saponins are natural surfactants with lipophilic aglycone and hydrophilic glycosyl groups. The aglycones of saponins composed of pentacyclic triterpenoids with oleanolic acid, and the glycosyl groups of saponins usually include glucose, galactose, rhamnose, xylose, etc. [23]. Differences in the aglycone structure and the glycosyl groups can be used to classify various saponins types. Saponins can be involved in some specific biological activities such as hemolysis [24]. They can combine with lipids and cholesterols on the surface of the red blood cells, making each ferrous ion complexed with four pyridine rings. Saponins can reduce the surface tension and dissolve in water easily due to their highly hydrophilic properties. Currently, only a few commercial saponins-containing products are available, most of which are derived from *Quillaja saponaria* and *Saponaria officinalis*. The rhizomes of the *Saponaria officinalis* plant contain more than 20% of saponins and have been used as detergents since ancient time [22]. Saponins in *Quillaja saponaria* are mainly triterpenes and their derivatives [25], which have been suggested as natural alternatives for the formation and stabilization of different dispersed systems due to their high surface activities. Saponins from *Saponaria officinalis* have a wide range of applications, such as surfactants and medical products [26]. Accordingly, the saponins in *Quillaja saponaria* and *Saponaria officinalis* can be potential natural surfactants for nanoemulsion preparations.

Modern research has focused on finding solutions for delivering lipophilic active substances with poor water solubility in form of aqueous beverages [27]. Approaches such as encapsulating these active substances with a small amount of oil and delivering them via tunable low-oil concentration emulsion systems have been suggested [28,29]. In a previous study [30], we documented the use of plant extracts from rosemary, sage and thyme for inhibiting hydroperoxides and maintaining vitamin E levels in hempseed oil. In this study, we use plant extracts containing saponins from *Saponaria officinalis* and *Quillaja saponaria* for stabilization in a low-oil-content hempseed oil emulsion system. The results of droplet size distribution (droplet diameter v. number) obtained from dynamic light scattering (DLS) determined our system to call as a nanoemulsion. The presented results are milestones for the practical use of plant-based surfactants for stabilizing low-oil-content emulsion systems.

## 2. Results and Discussion

### 2.1. Spectroscopic Analysis of Raw Materials

**Figure 1:**
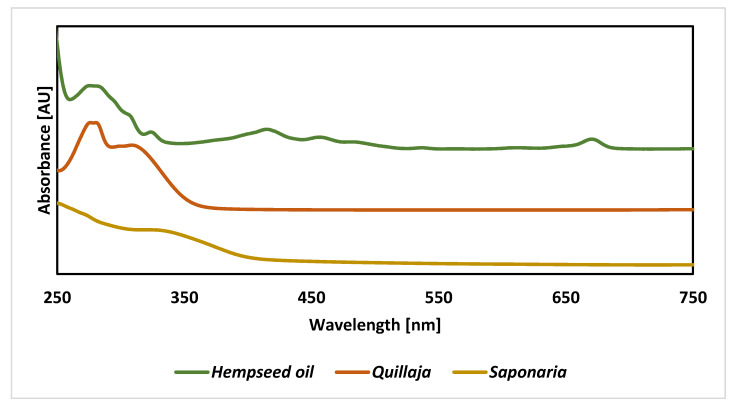
UV-Vis spectra of hempseed oil and 2 g/L water solutions of plant extracts from *Saponaria officinalis* and *Quillaja saponaria*.

Spectroscopic analyses were used to compare the chemical nature of the ingredients of the prepared emulsions. The analysis of spectra in the UV-Vis range shows relatively uniform spectra for plant surfactants. However, in the case of *Saponaria officinalis*, the main signal reached the maximum absorbance below 250 nm, with a pronounced inflection point at about 300 nm, while, for *Quillaja Saponaria*, the signal was split into two distinct signals with maxima at 280 and 310 nm (Figure 1). Based on literature, maximal absorbance below 250 nm is often attributed to phenol derivatives. The spectra of flavonoids and phenolic acids have clear signals at ca. 340 or 270 nm [31,32]. For example, flavonols, most commonly in the form of glycosides, show absorbance maxima in the wavelength range from 260 to 355 nm. Moreover, in the visible light range, there are no signals in the saponins spectrum, as confirmed by Reddy et al. [33], who performed spectra in the wavelength range from 400 to 800 nm for the aqueous solution of the *Sapindus mukorossi* extract, containing mainly saponins.

**Figure 2:**
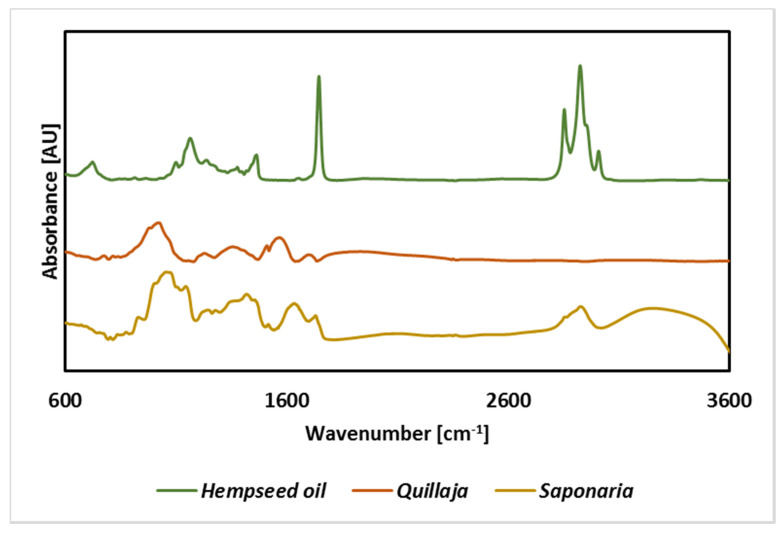
Infrared spectra of hempseed oil and used plant extracts from *Saponaria officinalis* and *Quillaja saponaria*.

Analysis of infrared spectra for the tested extracts confirmed the presence of bands characteristic of saponins, including C-H (3000–2800 cm^−1^) and C-O (approx. 1100 cm^−1^) (Figure 2). Signals from bands in the hydroxyl group (above 3000 cm^−1^) were only visible for the extract from *Saponaria officinalis*. Similar results for the spectra of extracts containing saponins were presented in the literature for the extract from the fruit of *S. mukorossi* [33] leaves of *Eclipta alba* [34] or seeds of *Cassia angustifolia*. It should be noted, however, that the signals observed in the spectrum of the saponins-containing extract may also be derived from bonds and groups of compounds that could be extracted simultaneously with saponins, such as flavonoids, phenol derivatives, saccharides, anthocyanins, steroid and triterpene compounds [35]. These compounds have many functional groups and bonds in common with saponins, which can translate into difficulties with a clear interpretation of the spectra obtained (Figure 2).

In turn, the analysis of spectra obtained for hempseed oil confirms the content of chlorophyll (signal at 675 nm) and conjugated C=C bonds (350–450 nm) in unsaturated fatty acids. A significant share of these bonds is confirmed by the infrared spectrum (signal about 3050 cm^−1^). This is a confirmation of literature data that show that this type of vegetable oil is characterized by a very high proportion of monounsaturated and unsaturated fatty acids. The crude oil also contains plant pigments, including carotenoids (signal at 470), and chlorophylls a and b (signal at 660–675) [36].

### 2.2. Particle Size Determination

**Figure 3:**
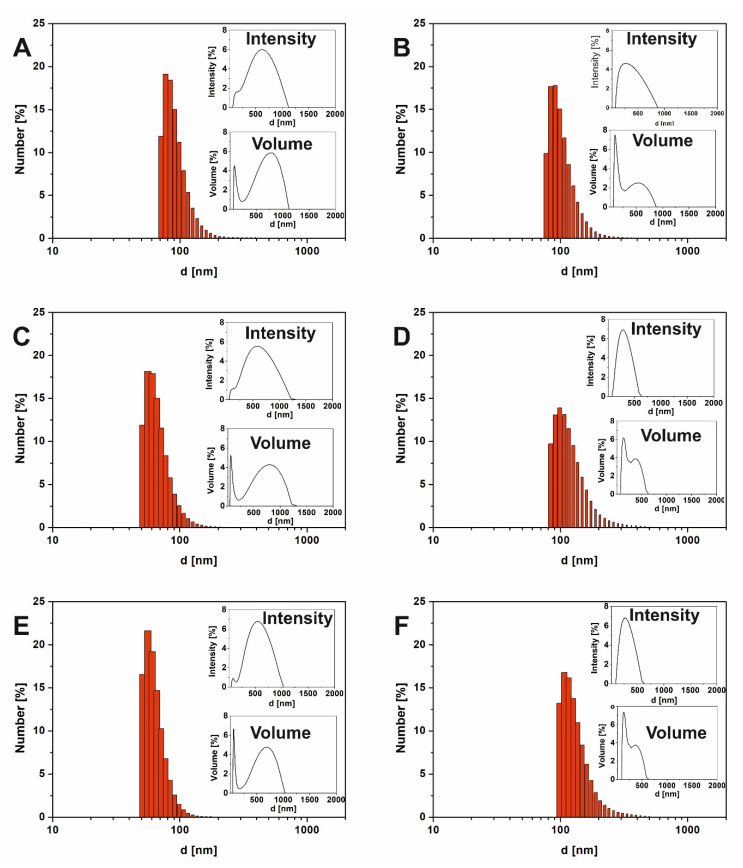
Droplet size distribution by number, intensity and volume obtained by dynamic light scattering (DLS): oil-in-water (O/W) without surfactant emulsion (**A**) after 24 h, (**B**) after 1 week; emulsion with *Quillaja* extract (**C**) after 24 h, (**D**) after 1 week; emulsion with *Saponaria* extract (**E**) after 24 h, (**F**) after 1 week.

Droplet size distribution is one of the parameters that describes emulsion systems and has an impact on their stability. One of the most frequently applied techniques for particle size distribution in emulsion systems including food emulsions is dynamic light scattering (DLS) [37]. Table 1 shows the results of particle size distributions for samples with constant oil concentration (2% *v*/*v*) but different concentrations of saponins (0–2 g/L). Both plant extracts possessed CMC equal to 1 g/L. The values of d-ave (intensity-based overall average size, determined by cumulant method) with main peaks maxima and Polydispersity Indexes (PDI) were compared. It was observed that the concentration of saponins had a significant effect on the particle size of the emulsion (*p* < 0.05). Although the initial emulsions containing 2 g/L saponins showed higher values for d-ave, the droplet size decreased significantly (*p* < 0.05) after 1 week of storage. The PDI were also reduced significantly (*p* < 0.05) from 26–27% to 21–26% after 1 week (Table 1). Considering these results as well as the emulsion stability tests (see p. 2.4.), we noticed that most stable systems with saponins-based surfactants were samples containing 2 g/L (2 CMC) of extract. Detailed studies (inset charts, described as “Intensity” on top right corners in Figure 3) showed that, for most of the samples, two main fractions were distinguished. The first fraction represented droplets with diameters lower than 100 nm, recording after 24 h of emulsification. In the same time, an intensive signal from droplets with diameters c.a. 450–520 nm were recorded (Figure 3A,C,E). For a longer storage time, the diameters of both fractions decreased. This effect was noticed in various nanoemulsion systems [38,39] as well as for hempseed oil nanoemulsions [13]. Badolato et al. [40] suggested that larger droplets refer to less stability of the emulsion system.

Based on our previous experience [10,41], we decided to present droplet size distribution by intensity, number and volume ratio (Figure 3). In our previous work [10] we suggested that during the DLS results analysis, the intensity of scattered light by particles should be considered for nanoparticles or droplet size characterization. One of the reasons for this is that the presence of even a few large particles (possible contaminations or samples with high PDI index) strongly affects the average particle’s diameter. One of the frequently used approaches for the elimination of this phenomena is the use of a filtration process. Unfortunately, additional processes might strongly affect the valuable information of the “real conditions” in the investigated samples. As a result, detailed particle/droplet size distribution evaluation, including the results for intensity and particle number versus particle size, were presented for the most stable samples (see Figure 3).

The DLS measurement analysis of the particle/droplet number versus droplet size showed that small droplets dominated in the whole volume, in each sample. The results showed that saponins were effective emulsion stabilizers, promoting oil dispersion in the aqueous phase. For instance, comparing reference sample (with no saponins) with samples containing 2 g/L saponins (Figure 3) as well as samples with different saponins origin (*Quillaja* and *Saponaria*), it is clear that the size of droplets in emulsion after 24 h of preparation was smaller for the nanoemulsions with saponins content (about 56 nm) than to that of samples without plant extracts (about 77 nm). Interestingly, according to droplet size distribution by number, the size of droplets in the reference sample was stable at least for a week.

The analysis of the particle size distribution by volume (see Figure 3) confirmed the presence of two main fractions. The plot presenting the droplets’ sizes by volume consists of two distinctive peaks for droplets with different diameters. The obtained data show that directly after emulsion preparation, large droplets (>500 nm in diameter) dominated all samples, regardless of the presence of extract and their concentration. This was accompanied by very wide distribution for large droplets and a sharp, narrow peak characterizing smaller ones. The relation of each peak contribution to the whole size distribution in those charts is related to active collisions (scattering) across the entire cross-section. Larger particles/droplets take much more space in the sample, which makes the collision/scattering phenomenon more probable; thus, larger droplets are more visible in such experiments.

### 2.3. Emulsion Stability

Completed studies have shown the high stability of the obtained emulsions based on saponins-rich plant extracts and hempseed oil. After 24 h from the time of homogenization, all samples showed homogeneity, completely constituting the emulsion layer. Hence, EI_24h_ (emulsification index determined after 24 h) can be taken as 100% for all samples, except the sample without saponins, when EI_24h_ was 97 ± 1% (Table 2). Visual observations of the samples made after 7 days showed a relatively clear separation into the bottom layer, notwithstanding the turbidity, which was more transparent than the strongly emulsified layer present on the surface of the sample. At the same time, the separation of the oil layer was observed in samples not containing plant extract and with a concentration equal to 0.5 g/L. It should be noted that an increase in the content of the highly emulsified layer, compared to samples without extracts, was noticeable at concentrations exceeding 1 g/L value. The more visible differences were noticeable for samples after 7 days of storage. In the control sample, without saponins, EI_7d_ dropped to 85%. The results for samples with plant extract have shown that the most beneficial for emulsion stability was the use of an extract from *Saponaria officinalis*, which allowed us to maintain a 98% emulsion layer at 2 g/L. For the extract of *Quillaja saponaria*, the content of the emulsion also reached the highest values (i.e., 90%) for a concentration of 2 g/L. Nevertheless, it should be underlined that at all tested concentrations, with more intensive turbid observed by eye was found in samples with *Saponaria* extract than with *Quillaja* extract.

Additional information was provided by sedimentation tests in which the emulsion separation process was accelerated due to centrifugation (Table 2). The elution layer was 3% in the sample without extract. The addition of both the extract from *Saponaria officinalis* and *Quillaja saponaria* caused the survival of the higher emulsion layer, reaching up to 11% at an extract concentration of 2 g/L. This value was not significantly changed (*p* > 0.05) for 5 h after centrifugation except for the sample with 0.5 g/L *Quillaja saponaria* extract. The high stability of emulsions obtained on the basis of hempseed oil has also been confirmed by studies by Jarzębski et al. [13], where pea protein was used as a stabilizer. Moreover, Kowalska et al. [42] found an EI_24h_ of 100% for emulsions with the same oil and carboxymethyl cellulose and lecithin as emulsifiers. So far, no studies have been conducted with plant extracts containing saponins as emulsifiers for hempseed oil. However, saponins have confirmed their high efficiency for emulsifying hydrocarbons (using *Saponaria* extract) [22] or vegetable oils, such as coconut, cottonseed, or palm oil (using *Quillaja* extract) [43].

**Figure 4:**
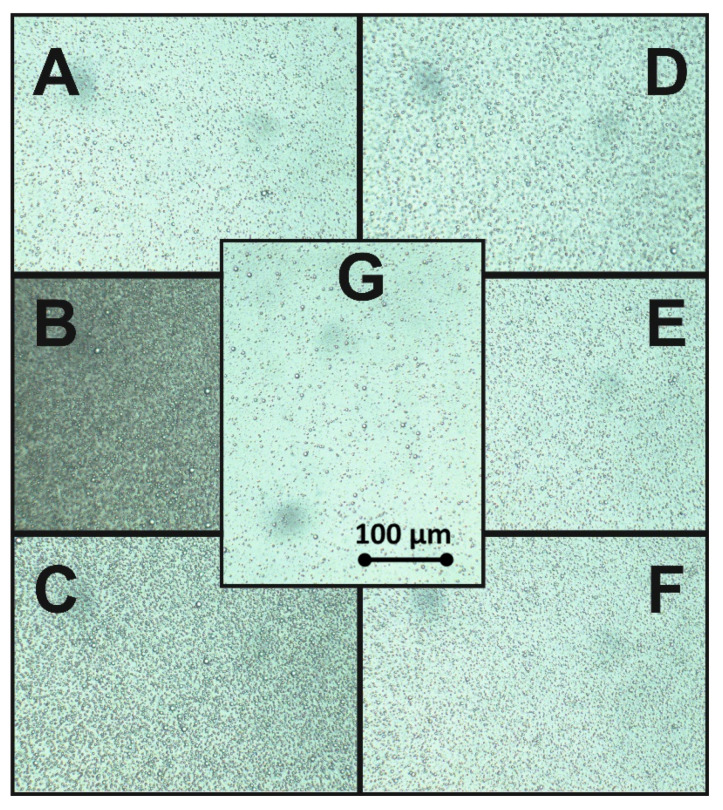
Microscopic images of hempseed oil (HSO) base emulsion with: *Quillaja* (**A**) 0.5 critical micelle concentration (CMC), (**B**) 1.0 CMC, (**C**) 2.0 CMC and *Saponaria* (**D**) 0.5 CMC, (**E**) 1.0 CMC, (**F**) 2.0 CMC and (**G**) the references sample O/W emulsion without surfactants.

An optical microscope was applied for the evaluation of the homogeneity of the samples with different concentarions of plant extract-derived surfactants. A high number of large droplets was observed in a light microscope in all samples (Figure 4A–G). No visible significant differences were observed in the reference sample, which was prepared without surfactant (Figure 4G). The uniformity of droplets might be caused lower oil concentration than in our previous research [44,45]. For detailed studies of droplet size and size distribution, we recommend using a confocal laser scanning microscope or scanning electron microscope with cryomode.

### 2.4. Surface and Interface Tension

**Figure 5:**
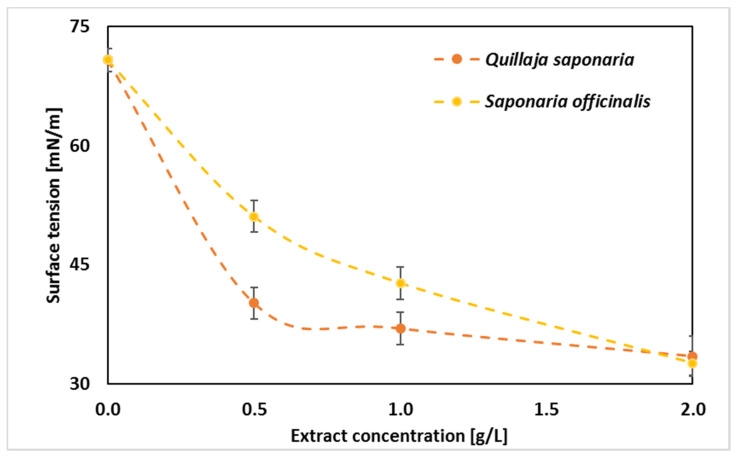
Surface tension versus surfactant concentration (SD presented as error bars).

Further research focused on assessing the surface-active properties of the emulsion ingredients can be found in Figure 5. Considering the impact of this parameter on the formation of emulsions, it should be noted that this is not the only parameter determining this phenomenon. However, the lower the surface tension, the easier the oil phase is to disperse in the aqueous phase, and the stability of such a system can also be assumed. In the presented experiment, the tension at the interface between the liquid and air phase (surface tension) and at the interface between the water and oil phases (interface tension) was determined. When applied at 2.0 g/L, the extract from *Saponaria officinalis* reduced the surface tension of the aqueous solution slightly more than the extract from *Quillaja saponaria* (32.6 mN/m compared to 33.5 mN/m). At lower concentrations, *Quillaja saponaria* extract (40.2 mN/m at 0.5 g/L, i.e., 0.5 CMC) proved to be significantly more effective at reducing surface tension. Similarly, the values of the interfacial tension, at the interface with oil, slightly differed from those mentioned above and reached approx. 42 mN/m. The surface tension of the emulsions were also at a similar level (approx. 49 mN/m). The last results are especially interesting because the measurements of the surface tension of complex mixture are not frequent in the literature. Nevertheless, emulsion processing and transport in an industrial installation, e.g., in dispensing nozzles, where surface tension allows for calculating, among others, capillary forces present in them. And the results show that the type of extract used does not cause significant differences in the surface tension of the resulting emulsions.

**Figure 6:**
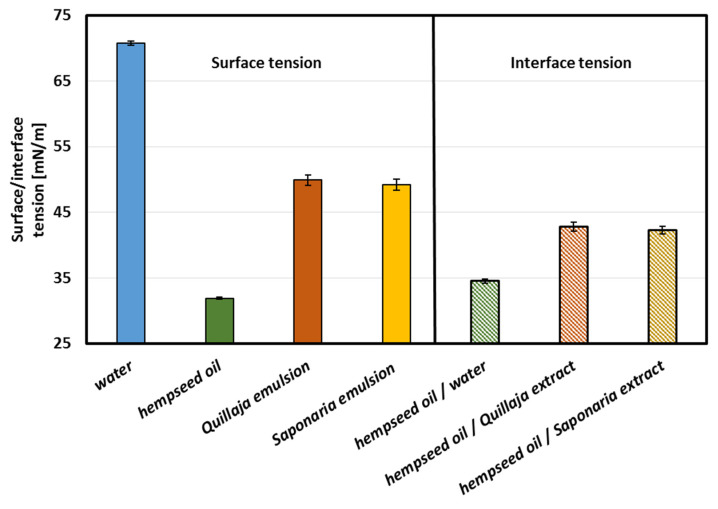
Surface tension of hempseed oil and water solutions of plant extracts from *Saponaria officinalis* and *Quillaja saponaria* at a concentration of 2 g/L and interface tension in the emulsion systems with and without surfactants.

Hempseed oil showed a relatively low surface tension of 32 mN/m (Figure 6). The last value is very similar to what was obtained by Esteban et al. [46], who measured surface tensions of different oils (rapeseed, corn, soybean, and sunflower oils) and found out that such surface tensions were equal to 31–33 mN/m. Moreover, Yang et al. [47] have shown that plant extract, such as *Quillaja saponaria* extract, contained comparable interface tensions (with Medium chain triglyceride oil–Miglyol 812) similar to commercial surfactant Tween 80. Changes in surface tension that were dependent on surfactant concentrations indicated their adsorption properties. Smułek et al. [22] calculated the free energy of adsorption as 29.7 kJ mol^−1^ and the area occupied by statistical surfactant molecule as 7.6 × 10^−19^ m^2^. For comparison, the value of the adsorption free energy for the compounds present in the extract of the *Gleditsia sinensis* determined by Wang et al. [48] was equal to −32.9 kJ mol^−1^, and the same parameter for *Sapindus mukorossi* extract was 41.4 kJ mol^−1^ [49]. Golemanov et al. [50] studied the surface properties of saponins’ solutions from *Quillaja saponaria*. The area occupied by the statistical particle was from 7.5 × 10^−19^ m^2^ to 2.6 × 10^−19^ m^2^, which is slightly lower than that for *Saponaria* saponins.

### 2.5. Emulsions’ Biological Activity

**Figure 7:**
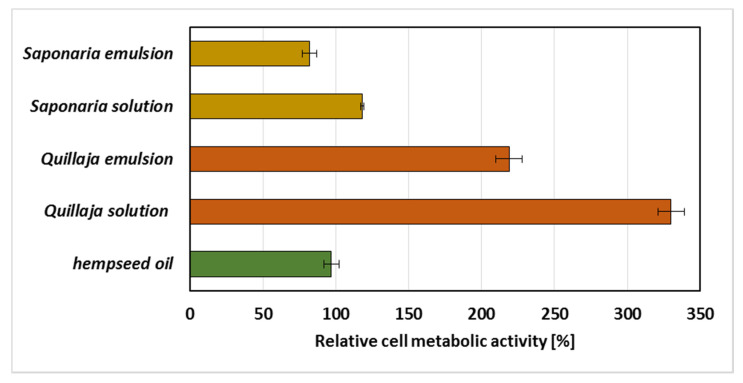
Relative metabolic activity of *Pseudomonas fluorescens* ATCC 17400 exposed to hempseed oil and water solutions and emulsions of plant extracts from *Saponaria officinalis* and *Quillaja saponaria*, with the metabolic activity in control sample set as 100% (SD presented as error bars).

The last part of the experiments included studies on the impact of the emulsion on representative microorganism, i.e., the Gram-negative bacterium *Pseudomonas fluorescens* ATCC 17400, which is a reference strain and representing a common bacterial species in the environment. For this purpose, the MTT reagent test, which allows one to determine the number of live cells and their metabolic activity condition, was used. The obtained results presented in Figure 7 show that the addition of hempseed oil, individually, did not strongly affect the measured parameter. In this sample, the metabolic activity was similar to that of the control (for which the activity value was 100%). All systems containing the extract of *Quillaja saponaria* showed increased biological activity, which may indicate that the components of the extract stimulated cell life processes. They could probably also be used as a source of energy for bacteria (Figure 7). Significantly lower metabolic activity was observed in samples containing the extract of *Saponaria officinalis*, which may suggest that the components of the extract showed slight toxic effects (in tests with emulsions, the metabolic activity dropped below 90%). In both series of samples with each of the extracts, the highest values of metabolic activity were found for systems without oil. Sen et al. [51] also observed that *Quillaja* extract enhanced the growth of other bacteria from the *Eschericha coli* strain. In contrast, Hassan et al. [52] noticed that *Quillaja* saponis were toxic against *Salmonella typhimurium*, *Staphylococcus aureus*. What is more, plant extracts are widely investigated as preservatives in foods because of their ability to inhibit bacteria growth [53,54]. Some authors reported bacteriostatic and antifungal properties depending on essential oils preparation [55,56,57]. Furthermore, Smułek et al. [22] investigated the toxicity of *Saponaria* extract and concluded that the extract was toxic against *Achromobacter* sp. SA1 bacteria cells (at concentration above 1 g/L) and *Pseudomonas putida* DA1 (at concentration above 2 CMC). Mikulcova et al. [8] studied hempseed oil emulsions stabilized by non-ionic surfactants (Tween 80 and 85, Span 80 and 85). They noticed that the samples showed antibacterial activity against *Bacillus*, *Salmonella* and *Serratia* strains, but not against *Eschericha coli*. This confirms the dependence between the bactericidal properties of saponins’ containing extracts as well as hempseed oil emulsions and the kind of the bacterial strain used in toxicity test.

## 3. Materials and Methods

### 3.1. Materials

#### 3.1.1. Reagents

All chemicals used, including inorganic salts, organic solvents, and reagents, were of analytical grade and were purchased from Sigma-Aldrich (Poznań, Poland). For all water solution preparation, Milli-Q water was used. The hempseed oil (cold pressed, unrefined) was purchased from Dary Natury (Grodzisk, Poland).

#### 3.1.2. Plant Extracts

To prepare the emulsion systems, two different plant extracts containing saponins were used. The first one is commercially available *Quillaja saponaria* bark extract (Sigma-Aldrich, Darmstadt, Germany). The second one is the *Saponaria officinalis* root (Flos, Mokrsko, Poland) extract, which was obtained and characterized as it was presented by Smułek et al. [22]. To evaluate total saponins content in the extracts, colorimetric method described by Hiai et al. [58] was used. The analysis showed that saponins content in *Quillaja* and *Saponaria* extract was 10 ± 2% and 5 ± 1%, respectively.

### 3.2. Emulsion Samples Preparation

A two-step process was employed to prepare the O/W nanoemulsions as described in our previous work [13] with some modifications. In brief, pre-emulsions were prepared using a homogenizer. In total, 2 mL of HSO was mixed with distilled water or extract solutions in distilled water up to a total volume of 50 mL (see Table 1). Then, the mixtures were vortexed for 10 min (5000 rpm) and directly thereafter homogenized for 10 min using an ultrasonicator in pulse mode (30 s/30 s), with a total energy of 4 kJ. To control the temperature during the ultrasonication process, all samples were kept in an ice bath. After the final preparation, the nanoemulsions were stored at 4 °C for further tests.

### 3.3. Characterization Methods

#### 3.3.1. Spectroscopic Analysis

The emulsion components were investigated using spectroscopic methods. The infrared spectra of freeze-dried plant extracts and hempseed oil were analyzed as KBr disks (1% of analyzed sample) using Fourier Transform Infrared Spectroscopy (FT-IR) spectrometer (Vertex 70, Bruker Daltonik GmbH, Bremen, Germany). The FTIR spectra of samples were collected at room temperature with a spectral range from 3600 to 600 cm^−1^ at a spectral resolution of 2 cm^−1^. Moreover, the UV-Vis spectra (spectral range from 250 to 750 nm) of hempseed oil and plant extract water solutions (at doubled critical micelle concentration, i.e., 2.0 g/L for *Saponaria* and *Quillaja* extract) were also obtained using a UV-Vis spectrophotometer (V-650, Jasco Inc., Tokyo, Japan).

#### 3.3.2. Dynamic Light Scattering

The size and volume of the droplets was evaluated by dynamic light scattering (DLS) using Litesizer^TM^ 500 by Anton Paar (Graz, Austria). Each sample measurement was repeated at least 6 times and the average values of diameter and PDI were calculated and presented in this manuscript. The temperature of measurements was set to 25 °C as close to room temperature and the equilibration time for the first measurement was 3 min. Each next measurement of the same sample was performed directly after the previous one, without any inspection. The measurements were performed using automatic regulation of laser beam (λ = 658 nm). The optics was adjusted for each measurement separately. The autocorrelation functions were registered for backscattered light at an angle of 175°.

#### 3.3.3. Emulsion Stability and Sedimentation Tests

The emulsification indexes were determined after 24 h (EI_24h_), and 7 days (EI_7d_). To that purpose, emulsions were stored in 10 mL plastic tubes at 20 °C. The emulsification index was calculated by measuring the ratio of emulsion layer height to total mixture height.

Sedimentation tests were carried out by centrifuging the obtained emulsions at 20 °C, 2500× *g* (3–18 K, Sigma Laborzentrifugen GmbH, Osterode am Harz, Germany) for 1 and 5 h, respectively.

#### 3.3.4. Microscopic Investigations

The morphology, homogeneity, and macroscopic structure of the obtained emulsions were studied using an optical microscope (BA410, Motic, Germany) equipped with a Moticam CCD color camera (Motic, Germany).

#### 3.3.5. Surface Tension Measurements

The equilibrium surface tension of the plant extract solutions, hempseed oil, and prepared emulsions was measured at 21 ± 1 °C by using the du Nouy platinum ring method with an Easy Dyne K20 tensiometer (Krüss, Hamburg, Germany).

### 3.4. Biological Tests

To evaluate the bioactive properties of the obtained emulsions, a microbial cells’ viability test was performed using 3-(4,5-dimethylthiazol-2-yl)-2,5-diphenyltetrazolium bromide assay (MTT) in accordance with the method described by Wang et al. [59]. The *Pseudomonas fluorescens* ATCC 17400 cells were cultivated for 24 h in nutrient broth, then centrifuged (4000× *g*, 10 min) and washed with mineral salt medium (composition (g/L): Na_2_HPO_4_∙2H_2_O 7.0, KH_2_PO_4_ 2.8, NaCl 0.5, NH_4_Cl 1.0). Afterwards, they were re-suspended in mineral salt medium to obtain an OD_600_ = 1.0. The research samples contained 0.1 mL of microbial suspension and 0.8 mL of emulsions and were incubated with 0.1 mL of 5 g/L MTT solution for 2 h at 30 °C. After incubation, the cultures were centrifuged at 15,000× *g*. Thereafter, the pellet (the formazan precipitate formed) was mixed with 1 mL of propan-2-ol, then the samples were centrifuged at 4000× *g*, and the supernatant was analyzed on a UV-Vis spectrophotometer at 560 nm. The higher concentration of the formed MTT formazan, the higher viability of bacterial cells in samples.

### 3.5. Statistical Analysis

The data of experiments are described as the mean ± SD of three tests. The data were statistically analyzed using Minitab software version 18.0 (State College, PA, USA). A one-way analysis of variance (ANOVA) followed by Tukey’s test as well as LSD were applied for analysis of data at *p* < 0.05.

## 4. Conclusions

The performed investigations confirmed the possible use of selected plant extracts containing saponins as a stabilizer for low oil-concentration hempseed oil emulsion. An amount of 2 g/L concentration of saponins’ content plant-based stabilizers helped in obtaining 100% emulsion stability, which decresed to only 98% and 90% after seven days of storage for *Saponaria* and *Quillaja* extracts, respectively. Accordingly, *Saponaria* extracts were found to be better than *Quillaja* extracts in terms of their emulsion stability, centrifugation stability, particle size and surface tension. Notably, *Quillaja* extracts were found to induce the growth of *Pseudomonas fluorescens ATCC 17400*, whereas *Saponaria* extracts depicted slight toxicity as they showed growth inhibition. Both *Quillaja* and *Saponaria* extracts were able to reduce the droplet size of the hempseed oil emulsions from c.a. 80 nm to 50 nm, as obtained by DLS after 24 h of emulsion preparation. The results of this study may provide some basic information for choosing plant-based extracts to obtain a stable emulsions system for the delivery of bioactives using hempseed oil as the carrier oil.

## Figures and Tables

**Table 1:** Emulsion droplet size values and Polydispersity Index (PDI) determined after 1 day and 1 week.

Origin of Saponins	Extract Content	After 1 Day				After 1 Week			
		d-ave	PDI	1st Peak Max	2nd Peak Max	d-ave	PDI	1st Peak Max	2nd Peak Max
	[g/L]	[nm]	[%]	[nm]	[nm]	[nm]	[%]	[nm]	[nm]
None	0	431 ± 17 ^Aa^	27.6 ± 1.5 ^A*i*^	520 ± 43	114 ± 22	254 ± 8 ^Cb^	23.5 ± 2.1 ^AB*ii*^	285 ± 11	42 ± 3
*Quillaja saponaria*	0.5	394 ± 15 ^Ba^	27.6 ± 1.5 ^A*i*^	503 ± 62	117 ± 27	279 ± 5 ^Bb^	25.8 ± 1.4 ^A*ii*^	325 ± 26	55 ± 1
	1	401 ± 12 ^Ba^	27.0 ± 1.6 ^A*i*^	473 ± 30	102 ± 83	304 ± 10 ^Ab^	25.1 ± 1.0 ^A*ii*^	344 ± 21	55 ± 15
	2	409 ± 18 ^ABa^	27.4 ± 1.4 ^A*i*^	500 ± 44	93 ± 25	247 ± 5 ^Cb^	23.4 ± 2.2 ^AB*ii*^	272 ± 16	34 ± 5
*Saponaria officinalis*	0.5	386 ± 11 ^Ca^	26.4 ± 1.1 ^A*i*^	428 ± 32	68 ± 12	256 ± 5 ^Cb^	22.0 ± 1.9 ^B*ii*^	276 ± 13	33 ± 1
	1	394 ± 15 ^Ba^	27.4 ± 1.2 ^A*i*^	451 ± 30	50 ± 1	249 ± 6 ^Cb^	23.3 ± 1.6 ^AB*ii*^	272 ± 17	28 ± 1
	2	437 ± 19 ^Aa^	26.3 ± 2.2 ^A*i*^	483 ± 18	79 ± 12	254 ± 6 ^Cb^	21.4 ± 2.2 ^B*ii*^	272 ± 18	45 ± 1

The results are expressed as mean ± standard deviation (*n* = 3); values on the same column that do not share the same uppercase letters are significantly different at *p* < 0.05; values on the same row for d-ave that do not share the same lowercase letters are significantly different at *p* < 0.05; values on the same row for PDI that do not share the same Roman numbers are significantly different at *p* < 0.05.

**Table 2:** Emulsification index after 24 h (EI_24h_) and 7 days (EI_7d_) of emulsion storage and emulsion content after sedimentation test with centrifuging at 2500× *g*.

Plant Extract	Extract Concentration [g/L]	EI_24h_ ± SD [%]	EI_7d_ ± SD [%]	Emulsion Layer Content ± SD after Centrifugation [%]	
				After 1 h	After 5 h
None	0	97 ± 1	85 ± 1	3 ± 1 ^a^	3 ± 1 ^a^
*Saponaria officinalis*	0.5	100 ± 0	89 ± 2	6 ± 1 ^a^	5 ± 2 ^a^
	1.0	100 ± 0	97 ± 1	8 ± 2 ^a^	8 ± 1 ^a^
	2.0	100 ± 0	98 ± 2	11 ± 2 ^a^	11 ± 2 ^a^
*Quillaja saponaria*	0.5	100 ± 0	85 ± 1	7 ± 1 ^a^	5 ± 1 ^b^
	1.0	100 ± 0	89 ± 1	8 ± 2 ^a^	8 ± 1 ^a^
	2.0	100 ± 0	90 ± 2	11 ± 1 ^a^	11 ± 2 ^a^

The values are the means± SD (*n* = 3); values on the same row with the same lowercase letters are not significantly different at *p* > 0.05.
